# Clonal Diversity and Resistome Dynamics of *Acinetobacter baumannii* Isolates from Lithuanian National Cancer Center

**DOI:** 10.3390/medicina61122151

**Published:** 2025-12-02

**Authors:** Tomas Liveikis, Danutė Labeikytė, Julija Armalytė, Kęstutis Sužiedėlis, Agnė Kirkliauskienė, Edita Sužiedėlienė

**Affiliations:** 1 Life Sciences Centre, Institute of Biosciences, Department of Biochemistry and Molecular Biology, Vilnius University, LT-10257 Vilnius, Lithuania; danute.labeikyte@gf.vu.lt (D.L.); julija.armalyte@gf.vu.lt (J.A.); kestutis.suziedelis@gf.vu.lt (K.S.); edita.suziedeliene@gf.vu.lt (E.S.); 2 Laboratory of Molecular Oncology, National Cancer Institute, LT-08406 Vilnius, Lithuania; 3 Microbiology and Laboratory Medicine, Faculty of Medicine, Institute of Biomedical Sciences, Department of Physiology, Biochemistry, Vilnius University, LT-03225 Vilnius, Lithuania; agne.kirkliauskiene@mf.vu.lt

**Keywords:** *A. baumannii*, tertiary oncology setting, antibiotic resistance, genotyping, biofilm

## Abstract

*Objectives:* To investigate the phenotypic and genotypic changes of *Acinetobacter baumannii* collected from the tertiary oncology setting in Lithuania. *Methods:* *A. baumannii* isolates (*n* = 61) were collected in the years 2013–2014 (*n* = 28) and 2017–2019 (*n* = 33) from a tertiary care cancer center in Lithuania. Antimicrobial susceptibility was determined according to EUCAST and for piperacillin/tazobactam and cefepime, according to CLSI guidelines. PCR, pulsed-field gel-electrophoresis, and multi-locus sequence typing were used for resistance gene detection and genotyping. The biofilm formation ability was determined by a microtiter plate assay. *Results:* Of 61 *A. baumannii* isolates obtained, 84% (51/61) and 71% (43/61) were multi-(MDR) and extensively (XDR) drug-resistant, respectively. Carbapenem-resistant isolates comprised 77% (47/61); of these, 92% (43/47) harbored genes encoding the OXA-23-like, and 4% (2/47) OXA-24-like carbapenemases. All isolates were susceptible to colistin. Genotyping analysis revealed six groups with the highest prevalence of international clones 1 (IC1) and 2 (IC2), which dominated during 2013–2014 and 2017–2019, respectively. Notably, the *A. baumannii* diversity increased in 2017–2019 with the emergence of 3-LST groups G4, G8, G12, and G14, which included isolates of ST276, ST78, ST1463, and ST1336 sequence types, respectively. The IC1 and IC2 isolates displayed characteristic gene profiles *aacC1*, *aacC2*, *aphA6*, *sul1*, and *armA*, *strA*-*strB*, *bla*_TEM_, respectively, whereas isolates from other groups had lesser resistance gene content. Isolates from IC2, G12, and G14 groups were strong biofilm producers; IC1, G4, and G8 isolates displayed no/weak biofilm formation capacity. *Conclusions:* *A. baumannii* from the cancer center showed a high prevalence of MDR and XDR phenotypes. Clonal dominance and diversity changed during the surveillance periods with the replacement of IC1 by IC2 clone isolates and the emergence of higher clonal diversity of isolates with stronger biofilm-forming capacity. The observed changes indicate a concerning trend of the establishment of a more virulent *A. baumannii* in the cancer setting.

## 1. Introduction

*Acinetobacter baumannii* is a Gram-negative, opportunistic pathogen commonly found in clinical environments worldwide and infects immunocompromised individuals in hospitals. *A. baumannii* causes a wide variety of infections, primarily nosocomial ventilator-associated pneumonia and bacteremia, urinary tract infections, wound infections, meningitis, and others [1]. Most of the isolates confer resistance to multiple groups of antibiotics, including last resort antibiotics such as carbapenems [2]. Cancer clinical units are at an especially high *A. baumannii* infection risk due to cancer patients having compromised immune systems, and a high potential of nosocomial transmission [3]. Cancer patients suffer from chemotherapy-induced co-morbidities such as neutropenia and damage of mucosal surfaces, which in turn increases *A. baumannii* infection risk [4].

The problem is exacerbated by the dissemination of several *A. baumannii* lineages, which are associated with multi-drug resistance (MDR) and clinical outbreaks globally, and by the ability to persist and spread in the clinical environment through phenotypic traits such as biofilm formation [5]. The ability of *A. baumannii* to survive on abiotic surfaces, such as catheters, endotracheal tubes, ventilators, and other medical equipment, favors its survival in the clinical environment and presents a significant barrier to infection control [6].

In Lithuania, *A. baumannii* is one of the most problematic pathogens causing hospital infections. According to European Centre for Disease Prevention and Control (ECDC) data, the proportion of carbapenem-resistant *Acinetobacter* spp. isolates in Lithuania has risen from 69.7% in 2014 to 92.6% in 2023 [7,8]. Moreover, the frequency of *Acinetobacter* spp. MDR isolates (resistant to fluoroquinolones, aminoglycosides, and carbapenems) [9] have increased from 60% to 83.1% during the same period [7,8].

Several studies have investigated antibiotic resistance and molecular features of *A. baumannii* in Lithuanian hospitals during different periods [10,11]. However, data on the genotypic and phenotypic characteristics of the *A. baumannii* population in cancer settings in Lithuania and neighboring countries are lacking [4]. Close epidemiological monitoring of this pathogen in cancer settings is warranted, as the rise of antibiotic-resistant *A. baumannii* and the rapid emergence of high-risk lineages pose a significant challenge to the treatment of infections in cancer patients. This study presents the first molecular characterization of *A. baumannii* isolates from a cancer care environment in Lithuania, highlighting the genotypic and phenotypic changes of strains that circulate in a tertiary oncology setting over time.

## 2. Materials and Methods

### 2.1. Bacterial Isolates

In this retrospective study, 61 non-duplicate *A. baumannii* isolates were collected at a tertiary care cancer center (National Cancer Center, Lithuania) with 270 beds between 2013 and 2014 (*n* = 28) and 2017 and 2019 (*n* = 33). The *A. baumannii*-positive cultures were recovered during routine clinical microbiology laboratory work from infections from hospitalized patients of all the wards of the cancer center, from wounds and pus (*n* = 21), respiratory tract (*n* = 16), urine (*n* = 9), abdomen (*n* = 9), catheter (*n* = 5), and other (*n* = 1). The identification of *A. baumannii* was done with the Matrix-Assisted Laser Desorption/Ionisation-Time-Of-Flight VITEK^®^ MS Microbial Identification System (bioMérieux, Marcy-l’Étoile, France), using VMS-P CE-IVD-certified reference database (version 3.2) and confirmed by PCR of the intrinsic *A. baumannii bla*_OXA-51-like_ gene [12]. Only *bla*_OXA-51-like_-positive isolates were included in the further analysis.

Recovered bacterial samples were grown overnight in Luria-Bertani (LB) medium at 37 °C. Cultures were mixed with glycerol 1:1 ratio and stored at −80 °C. Whenever applicable, bacteria are resuscitated on agarized LB medium at 37 °C overnight.

### 2.2. DNA Extraction

Preparation of lysates and DNA templates for PCR was obtained by the boiling method. Briefly, *A. baumannii* isolates stored at −80 °C were streaked on Luria-Bertani (LB) agar plate, and a single colony was suspended in 100 μL of sterile water, boiled for 10 min, and centrifuged at 10,000× *g* for 10 min. The supernatant was stored at −20 °C.

### 2.3. Antimicrobial Susceptibility Testing

Antimicrobial susceptibility testing was performed using the disk diffusion method on Muller-Hinton agar (Bio-Rad, Marnes-la-Coquette, France) according to the recommendations of the European Committee on Antimicrobial Susceptibility Testing (EUCAST) clinical breakpoints (v. 14.0) [13]. The susceptibility of all *A. baumannii* to gentamicin (10 μg), amikacin (30 μg), ciprofloxacin (5 μg), trimethoprim-sulfamethoxazole (1.25–23.75 μg), meropenem (10 μg), and imipenem (10 μg) was tested using commercial discs (Oxoid™, Basingstoke, UK). Susceptibility testing evaluations were performed for piperacillin/tazobactam and cefepime according to the CLSI (M100, 30th ed.). Colistin was evaluated using EUCAST breakpoint criteria. All three antibiotics were tested using the broth microdilution method (Liofilchem^®^, Roseto degli Abruzzi, Italy) [13,14]. For quality control, *Pseudomonas aeruginosa* ATCC 27853 and *Escherichia coli* NCTC 13846 were used (EUCAST v. 14.0). *A. baumannii* were categorised as MDR (non-susceptible to at least one agent in three or more categories of antimicrobials) and extensively drug resistant (XDR) (non-susceptible to at least one agent in all but two or fewer antimicrobial categories) according to Magiorakos et al. [9].

### 2.4. Identification of Antibiotic Resistance Genes

Antibiotic resistance genes were detected by PCR using primers listed in Supplementary Table S1. Reactions were performed according to the manufacturer’s recommendations, in a total volume of 12.5 µL, using DreamTaq PCR Master Mix (2X) (Thermo Fisher Scientific, Vilnius, Lithuania), 0.5 µL of each primer (final concentration 0.4 µM), and 1 µL of lysates as the DNA template. The PCR conditions were set as follows: initial denaturation at 95 °C for 5 min, 30 cycles, each cycle contained a denaturation step at 95 °C for 60 s, annealing at annealing temperature (Table S1) for 60 s, extension step at 72 °C for 60 s, and final extension for 7 min.

Fluoroquinolone resistance-conferring mutations were determined through restriction analysis of the *parC* and *gyrA* genes as described in [15].

### 2.5. Tri-Locus Sequence Typing (3-LST)

The tri-locus sequence typing method was used to assign international clonal groups to the isolates, as previously described [16,17]. Briefly, two multiplex-PCRs were performed using primers targeting alleles of *ompA*, *csuE*, and *bla*_OXA-51-like_ genes. Isolates were assigned to respective groups based on the different combinations of gene amplifications (Table S1).

### 2.6. Molecular Typing by Pulsed-Field Gel-Electrophoresis (PFGE)

The PFGE using *ApaI* restriction enzyme was performed as described by Povilonis et al. [10]. Results were analyzed with GelCompar II (version 6.5). Bands’ similarities were analysed and calculated using the Dice coefficient with 1.5% optimisation and 1% tolerance. Clusterisation method—Unweighted Pair Group Method with Arithmetic Mean, degeneracy cutoff value 80%. Results were grouped by similarity, and clusters were formed from isolates that were more than 80% similar.

### 2.7. Multi-Locus Sequence Typing

The Pasteur MLST scheme was used for typing [18]. The housekeeping genes *cpn60*, *fusA*, *gltA*, *pyrG*, *recA*, *rpoB*, and *rplB* of selected *A. baumannii* isolates were sequenced. The resulting sequences were analysed using the MLST database [19] to assign identified alleles to sequence types.

### 2.8. Biofilm Formation Assay

The biofilm assay was performed according to the method described by Yang et al. [20] in 96-well polystyrene plates. Mueller-Hinton broth without antibiotics was used as a negative control. The ability to form biofilm is classified according to the optical density cutoff (ODc), calculated as three standard deviations (SD) above the mean optical density (OD_530_) of the negative control. The ratio between the average optical density (OD) of the stained biofilm and the ODc was selected to represent the biofilm formation of each isolate. Three replicates were used for the biofilm assay.

### 2.9. Statistical Analysis

To calculate statistical significance, the Mann-Whitney test was used. A *p*-value of 0.05 was considered statistically significant.

## 3. Results

### 3.1. Isolate Genotyping

The 3LST method was used for *typing A. baumannii*. It allows clustering isolates into groups G1–14 [16], where G1, G2, and G3 correspond to IC2, IC1, and IC3 international clones, respectively [16]. Groups G4–14 do not have known equivalents to IC; however, they still allow for comparison of outbreak isolates between different studies, employing this typing scheme [16,17].

**Figure 1:**
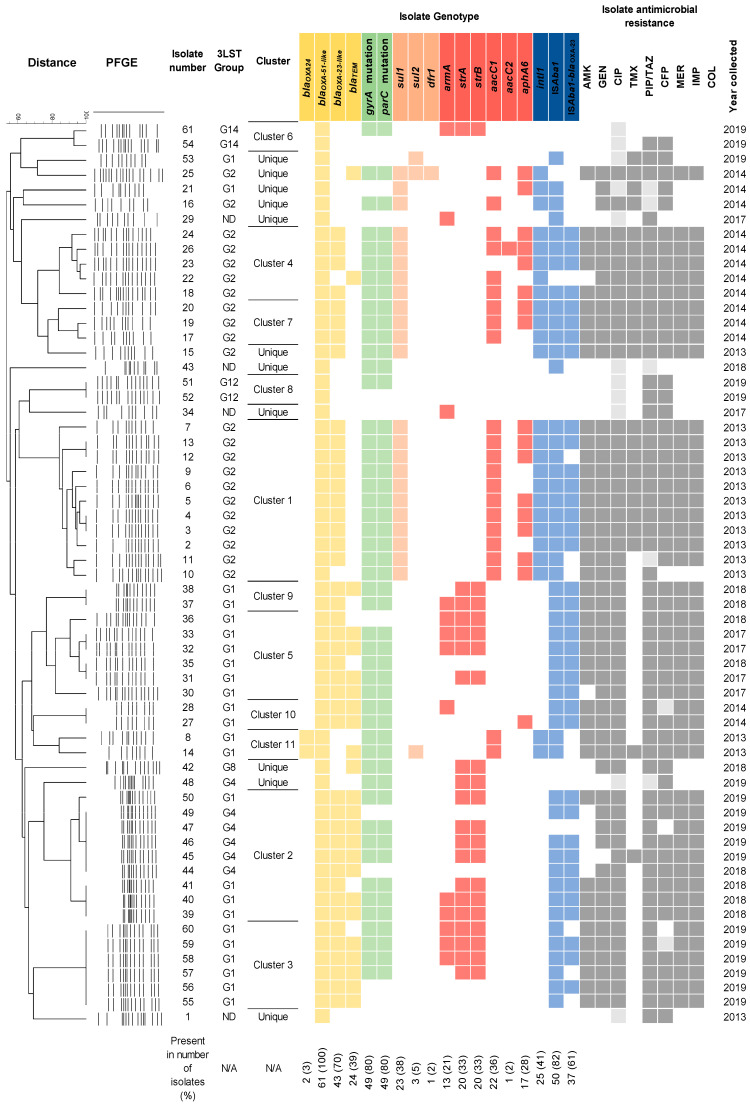
The heatmap showing *A. baumannii* genotypes and antibiotic susceptibility phenotypes. Antimicrobial resistance gene presence is indicated by color; white color indicates its absence. 3-LST column indicates group, assigned according to multiplex-PCR typing. ND—not determined. The percentage below the chart indicates the prevalence of the tested genes. A phylogenetic tree of PFGE-typed isolates is shown on the left. Black borders indicate clustered isolates that were more than 80% similar. The remaining isolates are considered unique. Antibiotic susceptibility phenotypes and year of isolation are shown on the right. Presence of genes conferring resistance to β-lactams, fluoroquinolones, sulfamethoxazole-trimethoprim, and aminoglycosides is indicated by yellow, green, orange, and red colors, respectively. Presence of integrons and insertion sequences is indicated by blue color. Resistant, intermediate resistant, and susceptible isolates are indicated by dark grey, light grey, and white colors, respectively.

A 3-LST typing revealed the presence of six PCR-based groups (G1, G2, G4, G8, G12, G14) (Figure 1). The majority of isolates fell into groups G1 (IC2) (39%, 24/61) and G2 (IC1) (36%, 22/61), respectively. The minor groups were G4 (10%, 6/61), G8 (3%, 2/61), and G12 and G14 (3% each, 2/61). IC1 isolates were common in 2013–2014, comprising 75% (21/28), and IC2 isolates accounted for 18% (5/28) (Figure 1). In the 2017–2019 period, IC2 comprised 58% (19/33) of isolates. Other isolates recovered in this period were assigned to G4 (6/33), G8 (1/33), G12 (2/33), and G14 (2/33) groups. The MLST typing of representative isolates from the G4 (#46 and #48), G8 (#42), G12 (#51), and G14 (#61) groups assigned them to sequence types ST2, ST267, ST78, and ST1336, respectively (Figure 1).

PFGE-*ApaI*-based genotyping has grouped isolates into eleven clusters by their similarity of at least 80% (Figure 1). Isolates belonging to clusters 3, 5, 9, 10, and 11 were assigned to the IC2 lineage. Isolates in clusters 1, 4, and 7 were assigned to IC1 lineage. Isolates grouped into clusters 1–5, 7, and 9–11 were carbapenem-resistant and possessed an MDR/XDR phenotype.

### 3.2. Antibiotic Susceptibility Profile

The antimicrobial susceptibility profiles revealed that MDR and XDR phenotypes were displayed by 84% (51/61) and 71% (43/61) of isolates, respectively. Carbapenem-resistant isolates accounted for 77% (47/61) of the isolates. All isolates from respiratory specimens and the abdomen were MDR, XDR, and CRAB. The occurrence of resistance phenotypes among *A. baumannii* recovered from other sites (wound and pus, urine, and catheters) varied, although over 70%, 40%, and 50% of isolates were MDR, XDR, and CRAB, respectively.

In 2013–2014, 86% (24/28) of isolates displayed CRAB phenotype, while in 2017–2019, 70% (23/33) of isolates were carbapenem-resistant. Of IC1 and IC2 isolates, 91% (20/22) and 79% (19/24) were carbapenem-resistant and exhibited either MDR/or XDR phenotypes, respectively. Similarly, isolates from 2013–2014 were more resistant to most antibiotics, with 93% (26/28) and 82% (23/28) displaying MDR and XDR phenotypes, respectively, compared to 76% (25/33) and 61% (20/33) of the isolates from 2017–2019. Almost a third of all isolates (31%, 19/61) displayed resistance to all tested antibiotics except colistin. High resistance rates were observed against ciprofloxacin (82%, 50/61), gentamicin (50%, 61/122), and amikacin (67%, 41/61). Of all isolates, 61% (37/61) were susceptible to trimethoprim-sulfamethoxazole, and 88% (21/24) of these belonged to IC2, which was recovered in both periods. Of all tested isolates, 11% (7/61) were susceptible to all antibiotics tested except ciprofloxacin. All isolates were susceptible to colistin (Table 1).

### 3.3. Antibiotic Resistance Genes

In total, 34 genes, conferring resistance against beta-lactams and carbapenems, aminoglycosides, fluoroquinolones, and sulfamethoxazole-trimethoprim were tested. Genes encoding β-lactamases *bla*_OXA-23-like_, *bla*_OXA-24-like_, and *bla*_TEM_ genes were found in 70% (43/61), 3% (2/61), and 39% (24/61) of isolates, respectively (Figure 1). Class 1 integrons were carried by 41% (25/61) of isolates recovered in 2013–2014, with most belonging to the IC1 lineage (86%, 22/25) (Figure 1). The majority of the isolates (82%, 50/61) contained the *IS*Aba1. Of the aminoglycoside resistance genes investigated, *aacC1* was the most common (36%, 22/61), followed by *strA-strB* (33%, 20/61) and *aphA6* (28%, 17/61), which provide resistance against gentamicin, streptomycin, and amikacin, respectively (Figure 1). The *armA* gene was found in 21% (13/61) of the isolates. With a few exceptions, *aacC1*, *aacC2* and *aphA6* genes (91%, 20/22, 5%, 1/22, and 68%, 15/22, respectively) were found in IC1 isolates from the 2013–2014 period, whereas *strA-strB* (58%, 14/24) and *armA* (42%, 10/24) genes were carried by IC2 isolates recovered in 2017–2019. Genes *qnrA*, *qnrS* and *aac(6‘)-Ib-cr* conferring resistance to quinolones were not found, whereas the presence of mutations in *gyrA* and *parC* genes rendering quinolone resistant phenotype [15] was observed in 80% (49/61) of isolates from both periods (Figure 1) with 2013–2014 period containing 93% (26/28) resistant isolates and 2017–2019 period, containing 70% (23/33) resistant isolates. Of the sulfamethoxazole and trimethoprim resistance genes tested, the most frequent was *sul1* (38%, 23/61), which correlated with class 1 integron carriage in three isolates. Less frequent were *sul2* (5%, 3/61) and *dfr1* (2%, 1/61) genes (Figure 1).

The non-IC1/2 isolates, representing the G4, G8, G12, and G14 groups, as well as unique isolates, most commonly carried blaTEM, strA, and strB, which were found in 40% (6/15) of the isolates, followed by blaOXA-23 in 33% (5/15). Mutations in *the parC* and *gyrA* genes were slightly more common in this group (53%, 8/15).

### 3.4. Biofilm-Forming Capability of A. baumannii Isolates

**Figure 2:**
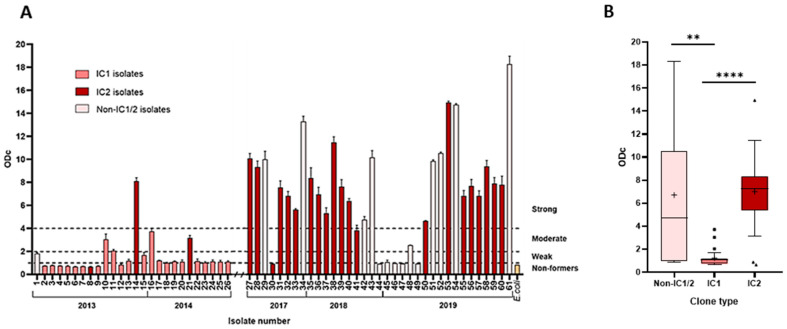
Biofilm formation of *A. baumannii* isolates. (**A**) The column chart depicts biofilm formation strength. The numbers below isolate numbers that denote the year of isolation. Dotted lines mark intervals of assigned biofilm formation strength; (**B**) The box-plot displays biofilm-forming capacity of IC1, IC2, and non-IC1/IC2 isolates. Measurements were done at 530 nm using safranin, as described in Methods. The whiskers extend 1.5 interquartile ranges, the median is displayed with a bar within the box, and the cross indicates the mean value. Black squares and triangles denote outliers. **—*p* < 0.01, ****—*p* < 0.0001.

The biofilm formation capacity of *A. baumannii* isolates has been evaluated. Of the studied isolates, 75% (46/61) were capable of producing biofilms. Of them, 46% (28/61) of isolates were categorised as strong biofilm producers, 10% (6/61) were moderate, 20% (12/61) were weak, and 25% (15/61) were non-producers (Figure 2A). IC2 lineage isolates were significantly stronger biofilm producers than IC1 isolates (*p* < 0.0001, Figure 2B). Most strong biofilm-producing IC2 isolates were XDR (80%, 16/20). Furthermore, non-IC1/2 isolates showed significantly (*p* < 0.01) stronger capacity to form biofilms compared to IC1 lineage isolates.

## 4. Discussion

This study aimed to characterize *A. baumannii* isolates collected in different periods from Lithuania’s largest tertiary care cancer setting. Our study reveals that a vast majority of *A. baumannii* isolates exhibited CRAB, MDR, and XDR profiles, respectively. The proportion of resistant isolates remained high during both periods under investigation.

While data about *A. baumannii* resistance in cancer settings are lacking, studies conducted in similar periods in other Lithuanian tertiary care medical centers reported that CRAB isolates comprised 43% of *A. baumannii* in 2010–2011 [10], whereas in 2016–2017 and 2021–2022 the incidence of CRAB exceeded 80% [10,11]. According to 2014 surveillance data, 70% of *Acinetobacter* spp. in Lithuania were found to be carbapenem-resistant [8]. The CRAB incidence in the oncology setting during a similar period was found to be even higher, indicating that a highly resistant *A. baumannii* population had already been present in healthcare settings a decade ago. According to our study, *A. baumannii* obtained during the investigated periods remained susceptible to colistin.

Comparing the situation with neighboring countries, similar to our findings, *A. baumannii* isolates collected in 2013 in hospitals in Poland showed a high resistance rate (80.8% XDR) to all antimicrobials except colistin [21]. The *A. baumannii* isolates recovered later period (2019–2021) in ICUs of hospitals in Poland were 86.6% XDR; the susceptibility to colistin remained high (92%) [22]. A study from Latvia found that over 70% of *Acinetobacter* spp. isolates obtained from the country’s largest hospital between 2017 and 2019 exhibited a CRAB phenotype, similar to our observations, and were colistin-susceptible [23]. *A. baumannii* resistance patterns in neighboring countries show similar trends, indicating a high regional resistance burden. Yet data from oncology hospitals in the region are scarce, despite cancer patients having a higher risk of *A. baumannii* infection [24,25]. Surveillance of *A. baumannii* resistance in cancer care settings is therefore important for comparing resistance profiles with non-oncology facilities and assessing the need for tailored infection-control or treatment strategies.

The results of this study revealed a change in the clonal population of *A. baumannii* in the oncology setting, showing an increasing prevalence of IC2 strains. The observed trend is in concordance with other studies, as described in *A. baumannii* strains from hospitals in Poland, where the dominant clonal types in 2013 were IC1 and IC2 [23]. In later periods (years 2019 and 2021), a shift towards the prevalence of IC2 was observed [23]. Similarly, prior studies have reported the dominance of IC1 and IC2 lineages in Lithuanian hospitals from 2010 to 2011, with frequencies of 52% and 45%, respectively [10]. However, the recent surveillance studies indicate the establishment of a high-risk IC2 lineage globally, with an approximately 80% incidence rate in Europe [26].

Importantly, we have identified several non-IC1/2 groups (G4, G8, G12, G14) that appeared in the 2017–2019 period, whose isolates represent ST types, some of which are being increasingly reported in Europe, such as the ST78 variant, found in this study in a G8 group isolate recovered in 2018. Most of the recently reported ST78 isolates are characterized by the production of *bla*_OXA-72_ carbapenemase and *bla*_CTX-M-115_ extended-spectrum β-lactamase [27,28]. The sporadic ST78 isolate identified in our study was susceptible to carbapenems and most of the antibiotics tested, except cefepime, and was intermediately resistant to ciprofloxacin and piperacillin/tazobactam. The carbapenem-susceptible ST78 isolates were reported in a 2011 study from Italy, which corresponded to 3-LST group G6, in contrast to our study, which assigned ST78 to G8 [28].

Other observed MLST types ST267, ST1463, and ST1336, representing isolates from G4, G12, and G14 groups, respectively, are rarely reported [29,30,31,32,33].

The investigation of the genes coding for carbapenem-hydrolyzing class D β-lactamases (CHDLs) in *A. baumannii* circulating in the cancer setting has shown the dominance of the *bla*_OXA-23-like_ gene. While CRAB isolates carrying the *bla*_OXA-23-like_ gene are disseminated worldwide [34], a previous study revealed that *bla*_OXA-24/40-like_ gene variant *bla*_OXA-72_ was the most frequent among CHDL-producing *A. baumannii* in Lithuanian hospitals [10]. This indicates the recent shift in the CHDL profile of *A. baumannii* isolates circulating in Lithuanian clinical settings. The prevalence of the CHDL profiles varies in neighboring countries and across the region. The trend observed in Portugal and Bulgaria aligns with our findings, where *bla*_OXA-23-like_ replaced *bla*_OXA-24/40-like_, which was the primary determinant of antimicrobial resistance in 2006 and 2008, respectively [35,36]. In contrast, the high prevalence of the *bla*_OXA-24/40-like_ variant, *bla*_OXA-72_, among *A. baumannii* has been reported in southern Poland [37]. These differences may be attributed to the dissemination type of *bla*_OXA-23_ and *bla*_OXA-24/40-like_, where *bla*_OXA-23_ is spread via transposon *Tn*2006, which can be located in both plasmids and chromosomes, while *bla*_OXA-24/40-like_ is commonly disseminated via clonal expansion and sometimes plasmids [38,39].

The analysis of *A. baumannii* resistance gene profiles has revealed an association with clonal lineages. The IC1 more frequently carried genes conferring resistance to aminoglycosides (*aacC1* and *aphA6*) and sulfonamides (*sul1*), whereas IC2 isolates had genes encoding OXA-23 carbapenemase and ArmA 16S rRNA methylase, commonly lacked sulfonamide resistance genes and integron structures, and showed susceptibility to trimethoprim/sulfamethoxazole (TMX). The co-carriage of *bla*_OXA-23_ and *armA* genes has been attributed to carbapenem and aminoglycoside resistance of IC2 lineage isolates [40]. Some studies have also found that *A. baumannii* clones carry *armA* or *bla*_OXA-23_ genes on plasmids, which represent a potential source for co-resistance dissemination [41,42]. The presence of the *armA* gene among *A. baumannii* isolates in Lithuanian clinical settings has been previously reported to be sporadic [41]. In contrast, this study revealed an increased prevalence of an MDR profile conferred by the co-production of ArmA and OXA-23 among IC2 isolates. The observed susceptibility of carbapenem- and aminoglycotherebyside-resistant IC2 isolates to TMX suggests that it may be considered a therapeutic option for treating MDR *A. baumannii* infections, given the current molecular epidemiology situation in the clinical setting. While TMX is not used as a first-line agent, some studies using this drug in combination therapy were successful [43]. It is worth noting that some isolates shared AMR determinants but differed phenotypically (Figure 1), likely due to untested efflux pumps, which may influence antimicrobial resistance [44].

The capacity of *A. baumannii* to form biofilms on various surfaces contributes to its survival in the clinical environment and antibiotic resistance, thereby challenging the treatment and control of infections [5]. Our study revealed that the *A. baumannii* population in a cancer setting shifted from non-producers or weak producers to the dominance of strong biofilm-producing isolates, primarily of the IC2 lineage. Moreover, the vast majority of IC2 biofilm-formers possessed an XDR phenotype. The IC2 is a high-risk *A. baumannii* clonal lineage causing nosocomial outbreaks worldwide [45]. Infections caused by the IC2 lineage of *A. baumannii* have been linked to high mortality rates [45,46]. The occurrence of IC2, exhibiting biofilm-forming and XDR phenotypes in the oncology setting, may significantly contribute to the long-term persistence of *A. baumannii*, thereby increasing the risk of contamination and colonization of cancer patients undergoing frequent invasive diagnostic and therapeutic procedures during treatment with chemotherapy, radiation, and surgery. This highlights the need for active surveillance in cancer settings, which involves monitoring the emergence of high-risk *A. baumannii* variants. Strict adherence to infection control practices is crucial in preventing the spread of this hospital-associated pathogen.

Some limitations of this study must be addressed. First, this study is limited to a single cancer center and may not accurately reflect the characteristics of *A. baumannii* in other cancer settings during the investigated period. Second, *A. baumannii* were not tested in 2015–2016, and some specific phenotypic and genotypic changes may have been missed. Third, the genotyping and gene detection methods used may not capture all gene and genotype variations; therefore, whole genome sequencing would allow for higher-resolution analysis.

## 5. Conclusions

In conclusion, our study shows significant changes in the clonal dominance and diversity of *A. baumannii* in the cancer clinical setting from 2013–2014 to 2017–2019. The trend towards an increasing frequency of IC2 isolates and the emergence of higher clonal diversity of isolates, which exhibit stronger biofilm-forming capacity, suggests the emergence of clones with enhanced persistence and virulence potential in *A. baumannii* in the cancer setting.

## Figures and Tables

**Table 1:** Antimicrobial susceptibility of *A. baumannii* isolates.

Antimicrobial Agent	Resistant, n (%)	Intermediate, n (%)	Susceptible, n (%)
Amikacin	41 (67)	0 (0)	20 (33)
Gentamicin	50 (82)	0 (0)	11 (18)
Trimethoprim/Sulfamethoxazole	24 (39)	0 (0)	37 (61)
Ciprofloxacin	50 (82)	11 (18)	0 (0)
Meropenem	47 (77)	0 (0)	14 (23)
Imipenem	47 (77)	0 (0)	14 (23)
Piperacillin/tazobactam *	55 (90)	5 (8)	1 (2)
Cefepime *	53 (87)	2 (3)	6 (10)
Colistin	0 (0)	0 (0)	61 (100)

*—The susceptibility breakpoints for piperacillin/tazobactam and cefepime were interpreted according to CLSI.

## Data Availability

The original contributions presented in this study are included in the article/Supplementary Materials. Further inquiries can be directed to the corresponding author.

## Supplementary Materials

The following supporting information can be downloaded at: https://www.mdpi.com/article/10.3390/medicina61122151/s1, Table S1: Primers used in this study. References [47,48,49,50,51,52,53,54,55,56,57,58,59,60,61,62,63,64,65] are mentioned in the Supplementary Materials.

## Abbreviations

The following abbreviations are used in this manuscript:

CLSI	Clinical & Laboratory Standards Institute
CRAB	Carbapenem-resistant *Acinetobacter baumannii*
ECDC	European Centre for Disease Prevention and Control
EUCAST	European Committee on Antimicrobial Susceptibility Testing
IC	International Clone
MDR	Multi-drug resistance
MLST	Muti-locus sequence typing
PFGE	Pulsed-field gel electrophoresis
ST	Sequence type
XDR	Extensive drug resistance
3-LST	Tri-locus sequence typing

## References

[1] Morris F.C., Dexter C., Kostoulias X., Uddin M.I., Peleg A.Y. (2019). The Mechanisms of Disease Caused by *Acinetobacter baumannii*. Front. Microbiol..

[2] Sharma R., Lakhanpal D. (2025). *Acinetobacter baumannii:* A Comprehensive Review of Global Epidemiology, Clinical Implications, Host Interactions, Mechanisms of Antimicrobial Resistance and Mitigation Strategies. Microb. Pathog..

[3] Freire M.P., de Oliveira Garcia D., Garcia C.P., Campagnari Bueno M.F., Camargo C.H., Kono Magri A.S.G., Francisco G.R., Reghini R., Vieira M.F., Ibrahim K.Y. (2016). Bloodstream Infection Caused by Extensively Drug-Resistant *Acinetobacter baumannii* in Cancer Patients: High Mortality Associated with Delayed Treatment Rather than with the Degree of Neutropenia. Clin. Microbiol. Infect..

[4] Ntim O.K., Awere-Duodu A., Osman A.-H., Donkor E.S. (2025). Antimicrobial Resistance of Bacterial Pathogens Isolated from Cancer Patients: A Systematic Review and Meta-Analysis. BMC Infect. Dis..

[5] Gedefie A., Demsis W., Ashagrie M., Kassa Y., Tesfaye M., Tilahun M., Bisetegn H., Sahle Z. (2021). *Acinetobacter baumannii* Biofilm Formation and Its Role in Disease Pathogenesis: A Review. Infect. Drug Resist..

[6] Donlan R.M. (2002). Biofilms: Microbial Life on Surfaces. Emerg. Infect. Dis..

[7] ECDC Antimicrobial Resistance in the EU/EEA (EARS-Net)—Annual Epidemiological Report 2023. https://www.ecdc.europa.eu/en/publications-data/antimicrobial-resistance-eueea-ears-net-annual-epidemiological-report-2023.

[8] ECDC Antimicrobial Resistance Surveillance in Europe 2014. https://www.ecdc.europa.eu/en/publications-data/antimicrobial-resistance-surveillance-europe-2014.

[9] Magiorakos A.-P., Srinivasan A., Carey R.B., Carmeli Y., Falagas M.E., Giske C.G., Harbarth S., Hindler J.F., Kahlmeter G., Olsson-Liljequist B. (2012). Multidrug-Resistant, Extensively Drug-Resistant and Pandrug-Resistant Bacteria: An International Expert Proposal for Interim Standard Definitions for Acquired Resistance. Clin. Microbiol. Infect..

[10] Povilonis J., Šeputienė V., Krasauskas R., Juškaitė R., Miškinytė M., Sužiedėlis K., Sužiedėlienė E. (2013). Spread of Carbapenem-Resistant *Acinetobacter baumannii* Carrying a Plasmid with Two Genes Encoding OXA-72 Carbapenemase in Lithuanian Hospitals. J. Antimicrob. Chemother..

[11] Černiauskienė K., Dambrauskienė A., Vitkauskienė A. (2023). Associations between β-Lactamase Types of *Acinetobacter baumannii* and Antimicrobial Resistance. Medicina.

[12] Turton J.F., Woodford N., Glover J., Yarde S., Kaufmann M.E., Pitt T.L. (2006). Identification of *Acinetobacter baumannii* by Detection of the blaOXA-51-like Carbapenemase Gene Intrinsic to This Species. J. Clin. Microbiol..

[13] EUCAST (2024). The European Committee on Antimicrobial Susceptibility Testing. https://www.eucast.org/bacteria/clinical-breakpoints-and-interpretation/clinical-breakpoint-tables/.

[14] (2017). Performance CLSI Standards for Antimicrobial Susceptibility Testing.

[15] Vila J., Ruiz J., Goñi P., Jimenez de Anta T. (1997). Quinolone-Resistance Mutations in the Topoisomerase IV parC Gene of *Acinetobacter baumannii*. J. Antimicrob. Chemother..

[16] Turton J.F., Gabriel S.N., Valderrey C., Kaufmann M.E., Pitt T.L. (2007). Use of Sequence-Based Typing and Multiplex PCR to Identify Clonal Lineages of Outbreak Strains of *Acinetobacter baumannii*. Clin. Microbiol. Infect..

[17] Karah N., Sundsfjord A., Towner K., Samuelsen Ø. (2012). Insights into the Global Molecular Epidemiology of Carbapenem Non-Susceptible Clones of *Acinetobacter baumannii*. Drug Resist. Updates.

[18] Diancourt L., Passet V., Nemec A., Dijkshoorn L., Brisse S. (2010). The Population Structure of *Acinetobacter baumannii*: Expanding Multiresistant Clones from an Ancestral Susceptible Genetic Pool. PLoS ONE.

[19] Jolley K.A., Bray J.E., Maiden M.C.J. (2018). Open-Access Bacterial Population Genomics: BIGSdb Software, the PubMLST.Org Website and Their Applications. Wellcome Open Res..

[20] Yang F., Liu C., Ji J., Cao W., Ding B., Xu X. (2021). Molecular Characteristics, Antimicrobial Resistance, and Biofilm Formation of *Pseudomonas Aeruginosa* Isolated from Patients with Aural Infections in Shanghai, China. Infect. Drug Resist..

[21] Chmielarczyk A., Pilarczyk-Żurek M., Kamińska W., Pobiega M., Romaniszyn D., Ziółkowski G., Wójkowska-Mach J., Bulanda M. (2016). Molecular Epidemiology and Drug Resistance of *Acinetobacter baumannii* Isolated from Hospitals in Southern Poland: ICU as a Risk Factor for XDR Strains. Microb. Drug Resist..

[22] Kasperski T., Romaniszyn D., Jachowicz-Matczak E., Pomorska-Wesołowska M., Wójkowska-Mach J., Chmielarczyk A. (2023). Extensive Drug Resistance of Strong Biofilm-Producing *Acinetobacter baumannii* Strains Isolated from Infections and Colonization Hospitalized Patients in Southern Poland. Pathogens.

[23] Jain N., Jansone I., Obidenova T., Simanis R., Meisters J., Straupmane D., Reinis A. (2021). Antimicrobial Resistance in Nosocomial Isolates of Gram-Negative Bacteria: Public Health Implications in the Latvian Context. Antibiotics.

[24] MacPhail A., Dendle C., Slavin M., McQuilten Z. (2024). Hospital-Acquired Bloodstream Infections in Patients with Cancer: Current Knowledge and Future Directions. J. Hosp. Infect..

[25] Grewal K., Sutradhar R., Krzyzanowska M.K., Redelmeier D.A., Atzema C.L. (2019). The Association of Continuity of Care and Cancer Centre Affiliation with Outcomes among Patients with Cancer Who Require Emergency Department Care. CMAJ.

[26] Müller C., Reuter S., Wille J., Xanthopoulou K., Stefanik D., Grundmann H., Higgins P.G., Seifert H. (2023). A Global View on Carbapenem-Resistant *Acinetobacter baumannii*. mBio.

[27] Higgins P.G., Hagen R.M., Podbielski A., Frickmann H., Warnke P. (2020). Molecular Epidemiology of Carbapenem-Resistant *Acinetobacter baumannii* Isolated from War-Injured Patients from the Eastern Ukraine. Antibiotics.

[28] Mammina C., Bonura C., Aleo A., Calà C., Caputo G., Cataldo M.C., Benedetto A.D., Distefano S., Fasciana T., Labisi M. (2011). Characterization of *Acinetobacter baumannii* from Intensive Care Units and Home Care Patients in Palermo, Italy. Clin. Microbiol. Infect..

[29] Ha V.N., Huy H.T., Đac T.N., Nguyen P.A., Cuong L.D. (2024). Genomic Epidemiology and Resistant Genes of *Acinetobacter baumannii* Clinical Strains in Vietnamese Hospitals. J. Med. Microbiol..

[30] Zhang P., Hao J., Zhang Y., Su J., Sun G., Xie J., Hu J., Li G. (2025). Understanding the Clinical and Molecular Epidemiological Characteristics of Carbapenem-Resistant *Acinetobacter baumannii* Infections within Intensive Care Units of Three Teaching Hospitals. Ann. Clin. Microbiol. Antimicrob..

[31] Muzahid N.H., Hussain M.H., Huët M.A.L., Dwiyanto J., Su T.T., Reidpath D., Mustapha F., Ayub Q., Tan H.S., Rahman S. (2023). Molecular Characterization and Comparative Genomic Analysis of *Acinetobacter baumannii* Isolated from the Community and the Hospital: An Epidemiological Study in Segamat, Malaysia. Microb. Genom..

[32] Nasser-Ali M., Aja-Macaya P., Conde-Pérez K., Trigo-Tasende N., Rumbo-Feal S., Fernández-González A., Bou G., Poza M., Vallejo J.A. (2024). Emergence of Carbapenemase Genes in Gram-Negative Bacteria Isolated from the Wastewater Treatment Plant in A Coruña, Spain. Antibiotics.

[33] Yusuf I., Idris H.B., Skiebe E., Wilharm G. (2025). Local Genomic Epidemiology of *Acinetobacter baumannii* Circulating in Hospital and Non-Hospital Environments in Kano, Northwest Nigeria. Curr. Microbiol..

[34] Lopes B.S., Amyes S.G.B. (2012). Role of ISAba1 and ISAba125 in Governing the Expression of blaADC in Clinically Relevant *Acinetobacter baumannii* Strains Resistant to Cephalosporins. J. Med. Microbiol..

[35] Domingues R., Oliveira R., Silva S., Araújo D., Almeida C., Cho G.-S., Franz C.M.A.P., Saavedra M.J., Azeredo J., Oliveira H. (2024). Molecular Detection of Carbapenemases in *Acinetobacter baumannii* Strains of Portugal and Association with Sequence Types, Capsular Types, and Virulence. Clin. Ther..

[36] Abbaszadeh F., Hasani A., Rezaee M.A., Sadeghi J., Hasani A., Oskouee M.A., Vahhabi A. (2021). Genetic Characterization of Extensive Drug Resistant *Acinetobacter baumannii*: An Appalling Impediment. Folia Medica.

[37] Jachowicz-Matczak E., Wołkowicz T., Kujawska A., Pałka A., Gajda M., Żółtowska B., Zacharczuk K., Piekarska K., Kasprzyk J., Wieczorek N. (2025). Epidemiological and Genomic Characterization of Carbapenem-Resistant *Acinetobacter baumannii* ST600 Harbouring the *bla*NDM-1 Gene, First Report in Poland. J. Glob. Antimicrob. Resist..

[38] Kuo S.-C., Huang W.-C., Huang T.-W., Wang H.-Y., Lai J.-F., Chen T.-L., Lauderdale T.-L. (2018). Molecular Epidemiology of Emerging blaOXA-23-Like- and blaOXA-24-Like-Carrying *Acinetobacter baumannii* in Taiwan. Antimicrob. Agents Chemother..

[39] Lee H.-Y., Chang R.-C., Su L.-H., Liu S.-Y., Wu S.-R., Chuang C.-H., Chen C.-L., Chiu C.-H. (2012). Wide Spread of Tn2006 in an AbaR4-Type Resistance Island among Carbapenem-Resistant *Acinetobacter baumannii* Clinical Isolates in Taiwan. Int. J. Antimicrob. Agents.

[40] Findlay J., Nordmann P., Bouvier M., Kerbol A., Poirel L. (2023). Dissemination of ArmA- and OXA-23-Co-Producing *Acinetobacter baumannii* Global Clone 2 in Switzerland, 2020–2021. Eur. J. Clin. Microbiol. Infect. Dis..

[41] Strateva T., Markova B., Marteva-Proevska Y., Ivanova D., Mitov I. (2012). Widespread Dissemination of Multidrug-Resistant *Acinetobacter baumannii* Producing OXA-23 Carbapenemase and ArmA 16S Ribosomal RNA Methylase in a Bulgarian University Hospital. Braz. J. Infect. Dis..

[42] Sánchez-Urtaza S., Ocampo-Sosa A., Rodríguez-Grande J., El-Kholy M.A., Shawky S.M., Alkorta I., Gallego L. (2024). Plasmid Content of Carbapenem Resistant *Acinetobacter baumannii* Isolates Belonging to Five International Clones Collected from Hospitals of Alexandria, Egypt. Front. Cell. Infect. Microbiol..

[43] Falagas M.E., Vardakas K.Z., Roussos N.S. (2015). Trimethoprim/Sulfamethoxazole for *Acinetobacter* Spp.: A Review of Current Microbiological and Clinical Evidence. Int. J. Antimicrob. Agents.

[44] Pană A.-G., Șchiopu P., Țoc D.A., Neculicioiu V.S., Butiuc-Keul A., Farkas A., Dobrescu M.-Ștefan, Flonta M., Costache C., Szász I.É. (2025). Clonality and the Phenotype–Genotype Correlation of Antimicrobial Resistance in *Acinetobacter baumannii* Isolates: A Multi-center Study of Clinical Isolates from Romania. Microorganisms.

[45] Castanheira M., Mendes R.E., Gales A.C. (2023). Global Epidemiology and Mechanisms of Resistance of *Acinetobacter baumannii*-Calcoaceticus Complex. Clin. Infect. Dis..

[46] Yu K., Zeng W., Xu Y., Liao W., Xu W., Zhou T., Cao J., Chen L. (2021). Bloodstream Infections Caused by ST2 *Acinetobacter baumannii*: Risk Factors, Antibiotic Regimens, and Virulence over 6 Years Period in China. Antimicrob. Resist. Infect. Control..

[47] Woodford N., Ellington M.J., Coelho J.M., Turton J.F., Ward M.E., Brown S., Amyes S.G.B., Livermore D.M. (2006). Multiplex PCR for Genes Encoding Prevalent OXA Carbapenemases in *Acinetobacter* Spp.. Int. J. Antimicrob. Agents.

[48] Yousfi K., Touati A., Lefebvre B., Garneau P., Brahmi S., Gharout-Sait A., Harel J., Bekal S. (2019). Characterization of Multidrug-Resistant Gram-Negative Bacilli Isolated from Hospitals Effluents: First Report of a blaOXA-48-like in *Klebsiella oxytoca*, Algeria. Braz. J. Microbiol..

[49] Dallenne C., Da Costa A., Decré D., Favier C., Arlet G. (2010). Development of a Set of Multiplex PCR Assays for the Detection of Genes Encoding Important β-Lactamases in Enterobacteriaceae. J. Antimicrob. Chemother..

[50] Poirel L., Walsh T.R., Cuvillier V., Nordmann P. (2011). Multiplex PCR for Detection of Acquired Carbapenemase Genes. Diagn. Microbiol. Infect. Dis..

[51] Garza-Ramos U., Morfin-Otero R., Sader H.S., Jones R.N., Hernández E., Rodriguez-Noriega E., Sanchez A., Carrillo B., Esparza-Ahumada S., Silva-Sanchez J. (2008). Metallo-β-Lactamase Gene blaIMP-15 in a Class 1 Integron, In95, from *Pseudomonas aeruginosa* Clinical Isolates from a Hospital in Mexico. Antimicrob. Agents Chemother..

[52] Maleki N., Tahanasab Z., Mobasherizadeh S., Rezaei A., Faghri J. (2018). Prevalence of CTX-M and TEM β-Lactamases *in Klebsiella pneumoniae* Isolates from Patients with Urinary Tract Infection, Al-Zahra Hospital, Isfahan, Iran. Adv. Biomed. Res..

[53] Hujer K.M., Hujer A.M., Hulten E.A., Bajaksouzian S., Adams J.M., Donskey C.J., Ecker D.J., Massire C., Eshoo M.W., Sampath R. (2006). Analysis of Antibiotic Resistance Genes in Multidrug-Resistant *Acinetobacter* Sp. Isolates from Military and Civilian Patients Treated at the Walter Reed Army Medical Center. Antimicrob. Agents Chemother..

[54] Wang A., Yang Y., Lu Q., Wang Y., Chen Y., Deng L., Ding H., Deng Q., Zhang H., Wang C. (2008). Presence of *qnr* Gene in *Escherichia coli* and *Klebsiella pneumoniae* Resistant to Ciprofloxacin Isolated from Pediatric Patients in China. BMC Infect. Dis..

[55] Doi Y., Arakawa Y. (2007). 16S Ribosomal RNA Methylation: Emerging Resistance Mechanism against Aminoglycosides. Clin. Infect. Dis..

[56] Wen J.-T., Zhou Y., Yang L., Xu Y. (2014). Multidrug-Resistant Genes of Aminoglycoside-Modifying Enzymes and 16S rRNA Methylases in *Acinetobacter Baumannii* Strains. Genet. Mol. Res..

[57] Gebreyes W.A., Altier C. (2002). Molecular Characterization of Multidrug-Resistant *Salmonella enterica* Subsp. *Enterica* Serovar Typhimurium Isolates from Swine. J. Clin. Microbiol..

[58] Aliakbarzade K., Farajnia S., Karimi Nik A., Zarei F., Tanomand A. (2014). Prevalence of Aminoglycoside Resistance Genes in *Acinetobacter baumannii* Isolates. Jundishapur J. Microbiol..

[59] Ramirez M.S., Tolmasky M.E. (2010). Aminoglycoside Modifying Enzymes. Drug Resist. Updat..

[60] Chen S., Zhao S., McDermott P.F., Schroeder C.M., White D.G., Meng J. (2005). A DNA Microarray for Identification of Virulence and Antimicrobial Resistance Genes in *Salmonella* Serovars and *Escherichia coli*. Mol. Cell. Probes.

[61] Vakulenko S.B., Donabedian S.M., Voskresenskiy A.M., Zervos M.J., Lerner S.A., Chow J.W. (2003). Multiplex PCR for Detection of Aminoglycoside Resistance Genes in Enterococci. Antimicrob. Agents Chemother..

[62] Randall L.P., Cooles S.W., Osborn M.K., Piddock L.J.V., Woodward M.J. (2004). Antibiotic Resistance Genes, Integrons and Multiple Antibiotic Resistance in Thirty-Five Serotypes of *Salmonella enterica* Isolated from Humans and Animals in the UK. J. Antimicrob. Chemother..

[63] Mak J.K., Kim M.-J., Pham J., Tapsall J., White P.A. (2009). Antibiotic Resistance Determinants in Nosocomial Strains of Multidrug-Resistant Acinetobacter Baumannii. J. Antimicrob. Chemother..

[64] Grape M., Motakefi A., Pavuluri S., Kahlmeter G. (2007). Standard and Real-Time Multiplex PCR Methods for Detection of Trimethoprim Resistance Dfr Genes in Large Collections of Bacteria. Clin. Microbiol. Infect..

[65] Koeleman J.G., Stoof J., Van Der Bijl M.W., Vandenbroucke-Grauls C.M., Savelkoul P.H. (2001). Identification of Epidemic Strains of *Acinetobacter baumannii* by Integrase Gene PCR. J. Clin. Microbiol..

