# Supplementary material for: Clonal Diversity and Resistome Dynamics of Acinetobacter baumannii Isolates from Lithuanian National Cancer Center
Source: Medicina (Kaunas). 2025 Dec 2;61(12):2151. doi: 10.3390/medicina61122151 (PMC12735066; doi:10.3390/medicina61122151)
Supplement: Supplementary file 1 [file medicina-61-02151-s001.zip › medicina-3981402-supplementary.pdf]

Supplements:

Table S1. Primers used in this study.

Gene	Primer sequences 5‘-3‘	Annealing temperature °C	Amplicon size (bp)	Reference
ompA_1	F:GATGGCGTAAATCGTGGTA<br>R:CAACTTTAGCGATTTCTGG	57	355	[16]
csuE_1	F:CTTTAGCAAACATGACCTACC<br>R:TACACCCGGGTTAATCGT	57	702	[16]
bla$_{OXA-66}$	F:GCGCTTCAAAATCTGATGTA<br>R:GCGTATATTTTGTTTCCATTC	57	559	[16]
ompA_2	F:GACCTTTCTTATCACAACGA<br>R:CAACTTTAGCGATTTCTGG	57	343	[16]
csuE_2	F:GGCGAACATGACCTATTT<br>R:CTTCATGGCTCGTTGGTT	57	580	[16]
bla$_{OXA-69}$	F:CATCAAGGTCAAACTCAA<br>R:TAGCCTTTTTTCCCCATC	57	162	[16]
bla$_{OXA-51-like}$	F:TAATGCTTTGATCGGCCTTG<br>R:TGGATTGCACTTCATCTTGG	55	353	[47]
bla$_{OXA-23-like}$	F:GATCGGATTGGAGAACCAGA<br>R:ATTTCTGACCGCATTTCCAT	54	1058	[47]
bla$_{OXA-58-like}$	F:AAGTATTGGGGCTTGTGCTG<br>R:CCCCTCTGCGCTCTACATAC	56	599	[47]
bla$_{OXA-24-like}$	F:GGTTAGTTGGCCCCCTTAAA<br>R:AGTTGAGCGAAAAGGGGATT	56	246	[48]
bla$_{VIM}$	F:GATGGTGTTTGGTCGCATA<br>R:CGAATGCGCAGCACCAG	54	390	[49]
bla$_{GIM}$	F:TCGACACACCTTGGTCTGAA<br>R:AACTTCCAACTTTGCCATGC	55	477	[50]
bla$_{SPM}$	F:AAAATCTGGGTACGCAAACG<br>R:ACATTATCCGCTGGAACAGG	55	271	[50]
bla$_{SIM}$	F:TACAAGGGATTCGGCATCG<br>R:TAATGGCCTGTTCCCATGTG	57	571	[50]
bla$_{IMP}$	F:GGAATAGAGTGGCTTAATTC<br>R:GCCAAGCTTCTATATTTGCG	50	275	[51]
bla$_{TEM}$	F:AGTATTCAACATTTCCGTGTCG<br>R:GCTTAATCAGTGAGGCACCTATC	57	851	[52]
gyrA	F:AAATCTGCCCGTGTCGTTGGT	59	343	[53]

	R:GCCATACCTACGGCGATACC			
parC	F:AAACCTGTTCAGCGCCGCATT<br>R:AAAGTTGTCTTGCCATTCACT	57	327	[53]
qnrA	F:ATTTCTCACGCCAGGATTTG<br>R:GATCGGCAAAGGTTAGGTCA	55	516	[54]
qnrS	F: ACGACATTCGTCAACTGCAA<br>R: TAAATTGGCACCCTGTAGGC	56	417	[54]
armA	F:ATTCTGCCTATCCTAATTGG<br>R:ACCTATACTTTATCGTCGTC	50	315	[55]
rmtB	F:GCTTTCTGCGGGCGATGTAA<br>R:ATGCAATGCCGCGCTCGTAT	59	173	[56]
strA	F:CTTGGTGATAACGGCAATTCC<br>R:CCAATCGCAGATAGAAGGCAA	59	547	[57]
strB	F:ATCGTCAAGGGATTGAAACCTA<br>R:GGATCGTAGAACATATTGGCG	57	510	[57]
aphA6	F:ATGGAATTGCCCAATATTATTC<br>R:TCAATTCAATTCATCAAGTTTTA	51	797	[58]
aacC1	F:AGCAGCAACGATGTTACGCA<br>R:CTGCGGGATCGTCACCGTA	58	470	[59]
aacC2	F:GGTTCGGCCTGCTGAATCA<br>R:AAGCCCACGACACCTTCTC	59	442	This study
aacC4	F:GATGGGCCACTTGGACTGAT<br>R:GCGCTCACAGCAGTGGTCAT	59	462	[60]
aadB	F:GAGCGAAATCTGCCGCTCTG<br>R:CTGTTACAACGGACTGGCC	58	320	[61]
sul1	F:TCACCGAGGACTCCTTCTTC<br>R:CAGTCCGCCTCAGCAATATC	57	331	[62]
sul2	F:CCTGTTTCGTCCGACACAGA<br>R:GAAGCGCAGCCGCAATTCAT	59	435	[62]
sul3	F:GATACAACTGAAGTGGGCGTT<br>R:ACGAGATTTCACATCGGTTCC	59	522	This study
folA	F:ATGGACAAAAATCACTGTATTGG<br>R:TAAGTGGCAAATTCGAATG	50	461	[63]
dfr1	F:TGGTAGCTATATCGAAGAATGGAGT<br>R:TATGTTAGAGGCGAAGTCTTGGGTA	58	425	[64]
dfr5	F:AGCTACTCTTTAAAGCCTTGACGTA<br>R:GTGTTGCTCAAAAACAACTTCG	58	341	[64]
dfr12	F:GAGCTGAGATATACACTCTGGCACT<br>R:GTACGGAATTACAGCTTGAATGGT	59	155	[64]

dfr17	F:ACATTTGACTCTATGGGTGTTCTTC<br>R:AAACTGTTCAAAAACCAAATTGAA	55	227	[64]
int1	F:CAGTGGACATAAGCCTGT<br>R:CCCGAGGCATAGACTGTA	53	160	[65]
int2	F:TTGCGAGTATCCATAACCTG<br>R:TTACCTGCACTGGATTAAGC	53	288	[65]
intl3	F:GCCTCCGGCAGCGACTTTCAG<br>R:ACGGATCTGCCAAACCTGACT	63	980	[65]

16. Turton, J.F.; Gabriel, S.N.; Valderrey, C.; Kaufmann, M.E.; Pitt, T.L. Use of Sequence-Based Typing and Multiplex PCR to Identify Clonal Lineages of Outbreak Strains of Acinetobacter Baumannii. *Clinical Microbiology and Infection* **2007**, *13*, 807–815, doi:10.1111/j.1469-0691.2007.01759.x.
47. Woodford, N.; Ellington, M.J.; Coelho, J.M.; Turton, J.F.; Ward, M.E.; Brown, S.; Amyes, S.G.B.; Livermore, D.M. Multiplex PCR for Genes Encoding Prevalent OXA Carbapenemases in Acinetobacter Spp. *Int J Antimicrob Agents* **2006**, *27*, 351–353, doi:10.1016/j.ijantimicag.2006.01.004.
48. Yousfi, K.; Touati, A.; Lefebvre, B.; Garneau, P.; Brahmi, S.; Gharout-Sait, A.; Harel, J.; Bekal, S. Characterization of Multidrug-Resistant Gram-Negative Bacilli Isolated from Hospitals Effluents: First Report of a blaOXA-48-like in Klebsiella Oxytoca, Algeria. *Braz J Microbiol* **2019**, *50*, 175–183, doi:10.1007/s42770-018-0010-9.
49. Dallenne, C.; Da Costa, A.; Decré, D.; Favier, C.; Arlet, G. Development of a Set of Multiplex PCR Assays for the Detection of Genes Encoding Important β-Lactamases in Enterobacteriaceae. *Journal of Antimicrobial Chemotherapy* **2010**, *65*, 490–495, doi:10.1093/jac/dkp498.
50. Poirel, L.; Walsh, T.R.; Cuvillier, V.; Nordmann, P. Multiplex PCR for Detection of Acquired Carbapenemase Genes. *Diagnostic Microbiology and Infectious Disease* **2011**, *70*, 119–123, doi:10.1016/j.diagmicrobio.2010.12.002.
51. Garza-Ramos, U.; Morfin-Otero, R.; Sader, H.S.; Jones, R.N.; Hernández, E.; Rodriguez-Noriega, E.; Sanchez, A.; Carrillo, B.; Esparza-Ahumada, S.; Silva-Sanchez, J. Metallo-β-Lactamase Gene blaIMP-15 in a Class 1 Integron, In95, from Pseudomonas Aeruginosa Clinical Isolates from a Hospital in Mexico. *Antimicrob Agents Chemother* **2008**, *52*, 2943–2946, doi:10.1128/AAC.00679-07.
52. Maleki, N.; Tahanasab, Z.; Mobasherizadeh, S.; Rezaei, A.; Faghri, J. Prevalence of CTX-M and TEM β-Lactamases in Klebsiella Pneumoniae Isolates from Patients with Urinary Tract Infection, Al-Zahra Hospital, Isfahan, Iran. *Adv Biomed Res* **2018**, *7*, 10, doi:10.4103/abr.abr_17_17.
53. Hujer, K.M.; Hujer, A.M.; Hulten, E.A.; Bajaksouzian, S.; Adams, J.M.; Donskey, C.J.; Ecker, D.J.; Massire, C.; Eshoo, M.W.; Sampath, R.; et al. Analysis of Antibiotic Resistance Genes in Multidrug-Resistant Acinetobacter Sp. Isolates from Military and Civilian Patients Treated at the Walter Reed Army Medical Center. *Antimicrob Agents Chemother* **2006**, *50*, 4114–4123, doi:10.1128/AAC.00778-06.
54. Wang, A.; Yang, Y.; Lu, Q.; Wang, Y.; Chen, Y.; Deng, L.; Ding, H.; Deng, Q.; Zhang, H.; Wang, C.; et al. Presence of Qnr Gene in Escherichia Coli and Klebsiella Pneumoniae Resistant to Ciprofloxacin Isolated from Pediatric Patients in China. *BMC Infect Dis* **2008**, *8*, 68, doi:10.1186/1471-2334-8-68.
55. Doi, Y.; Arakawa, Y. 16S Ribosomal RNA Methylation: Emerging Resistance Mechanism against Aminoglycosides. *Clinical Infectious Diseases* **2007**, *45*, 88–94, doi:10.1086/518605.

56. Wen, J.-T.; Zhou, Y.; Yang, L.; Xu, Y. Multidrug-Resistant Genes of Aminoglycoside-Modifying Enzymes and 16S rRNA Methylases in Acinetobacter Baumannii Strains. *Genet Mol Res* **2014**, *13*, 3842–3849, doi:10.4238/2014.May.16.9.
57. Gebreyes, W.A.; Altier, C. Molecular Characterization of Multidrug-Resistant Salmonella Enterica Subsp. Enterica Serovar Typhimurium Isolates from Swine. *Journal of Clinical Microbiology* **2002**, *40*, 2813–2822, doi:10.1128/jcm.40.8.2813-2822.2002.
58. Aliakbarzade, K.; Farajnia, S.; Karimi Nik, A.; Zarei, F.; Tanomand, A. Prevalence of Aminoglycoside Resistance Genes in Acinetobacter Baumannii Isolates. *Jundishapur J Microbiol* **2014**, *7*, e11924, doi:10.5812/jjm.11924.
59. Ramirez, M.S.; Tolmasky, M.E. Aminoglycoside Modifying Enzymes. *Drug Resist Updat* **2010**, *13*, 151–171, doi:10.1016/j.drup.2010.08.003.
60. Chen, S.; Zhao, S.; McDermott, P.F.; Schroeder, C.M.; White, D.G.; Meng, J. A DNA Microarray for Identification of Virulence and Antimicrobial Resistance Genes in Salmonella Serovars and Escherichia Coli. *Mol Cell Probes* **2005**, *19*, 195–201, doi:10.1016/j.mcp.2004.11.008.
61. Vakulenko, S.B.; Donabedian, S.M.; Voskresenskiy, A.M.; Zervos, M.J.; Lerner, S.A.; Chow, J.W. Multiplex PCR for Detection of Aminoglycoside Resistance Genes in Enterococci. *Antimicrob Agents Chemother* **2003**, *47*, 1423–1426, doi:10.1128/AAC.47.4.1423-1426.2003.
62. Randall, L.P.; Cooles, S.W.; Osborn, M.K.; Piddock, L.J.V.; Woodward, M.J. Antibiotic Resistance Genes, Integrons and Multiple Antibiotic Resistance in Thirty-Five Serotypes of Salmonella Enterica Isolated from Humans and Animals in the UK. *Journal of Antimicrobial Chemotherapy* **2004**, *53*, 208–216, doi:10.1093/jac/dkh070.
63. Mak, J.K.; Kim, M.-J.; Pham, J.; Tapsall, J.; White, P.A. Antibiotic Resistance Determinants in Nosocomial Strains of Multidrug-Resistant Acinetobacter Baumannii. *Journal of Antimicrobial Chemotherapy* **2009**, *63*, 47–54, doi:10.1093/jac/dkn454.
64. Grape, M.; Motakefi, A.; Pavuluri, S.; Kahlmeter, G. Standard and Real-Time Multiplex PCR Methods for Detection of Trimethoprim Resistance *Dfr* Genes in Large Collections of Bacteria. *Clinical Microbiology and Infection* **2007**, *13*, 1112–1118, doi:10.1111/j.1469-0691.2007.01807.x.
65. Koeleman, J.G.; Stoof, J.; Van Der Bijl, M.W.; Vandenbroucke-Grauls, C.M.; Savelkoul, P.H. Identification of Epidemic Strains of Acinetobacter Baumannii by Integrase Gene PCR. *J Clin Microbiol* **2001**, *39*, 8–13, doi:10.1128/JCM.39.1.8-13.2001.
